# Efficacy of Intrauterine infusion of granulocyte colony stimulating factor on patients with history of implantation failure: A randomized control trial

**Published:** 2016-11

**Authors:** Maryam Eftekhar, Sepideh Miraj, Maryam Farid Mojtahedi, Nosrat Neghab

**Affiliations:** 1 *Research and Clinical Center for Infertility, Shahid Sadoughi University of Medical Sciences, Yazd, Iran.*; 2 *Department of Obstetrics and Gynecology, School of Medicine, Shahrekord University of Medical Sciences, Shahrekord, Iran.*; 3 *Department of Obstetrics and Gynecology, Endocrinology and Female Infertility Unit, Roointan Arash Women’s Health Research and Educational Hospital, Tehran University of Medical Sciences, Tehran, Iran.*

**Keywords:** *GCSF*, *Pregnancy rate*, *In vitro fertilization*

## Abstract

**Background:**

Although pregnancy rate in in vitro fertilization-embryo transfer (IVF-ET) cycles has been increased over the preceding years, but the majority of IVF-ET cycles still fail. Granulocyte colony stimulating factor (GCSF) is a glycoprotein that stimulates cytokine growth factor and induces immune system which may improve pregnancy rate in women with history of implantation failure.

**Objective:**

The aim of this study was to evaluate GCSF ability to improve pregnancy rate in women with history of implantation failure

**Materials and Methods:**

0.5 ml (300 µg/ml) GCSF was infused intrauterine in intervention group. Pregnancy outcomes were assessed based on clinical pregnancy.

**Results:**

The mean age of participants was 31.95±4.71 years old. There were no significant differences between demographic characteristics in two groups (p>0.05). The pregnancy outcome in GCSF group was improved significantly (p=0.043).

**Conclusion:**

GCSF can improve pregnancy outcome in patients with history of implantation failure.

## Introduction

Although both subclinical and clinical pregnancy losses occur, the majority of failed IVF-ET cycles exhibit lack of implantation. Implantation failure may occur repeatedly. These patients are challenges for the infertility specialist (1, 2). Endometrial function and receptive endometrium are essential for implantation (3, 4). Repeated implantation failure (RIF) was defined as pregnancy failure after 2-6 times with at least 10 high quality embryos transferred into the uterus (5). 

About 40% of IVF cycles will be failed following one IVF cycle. IVF Failure is caused by either failure in early stage of implantation or early abortion. Major causes of IVF failure may be related to embryo quality and implantation failure (6-11). Granulocyte colony stimulating factor (GCSF) is synthesized in the reproductive tract naturally. GCSF stimulate neutrophilic granulocyte proliferation and differentiation, which act on macrophages of decidual cells, and finally affect implantation (12-14). 

The recruitment of dendritic cells, Th-2 cytokine secretion and activation of T regulatory cells are the others immune effects of GCSF. The cytokine increase the proportion of embryos that develop to the blastocyst stage from 30-76%. Also GCSF supplemented to the embryo culture medium improves implantation rate. Combination of follicular GCSF and IL-15 is an effective method to define oocyte component for successful conception in IVF/ICSI cycles (15-17). 

Most important role of GCSF is improving blastocyst development, aid to embryo implantation and improving devolvement of fetus and placenta (15, 16). GCSF can be effective treatment in patient with history of implantation failure and can improve endometrium growth. Also GCSF may increase pregnancy rate especially in women with thin endometrium (18, 19). GCSF is a glycoprotein that stimulates cytokine growth factor inducing immune system. GCSF stimulates the proliferation and differentiation of endometrial cells by CAMP increase in stromal cell through paracrine and autocrine signaling pathways (20). 

The aim of this study was to evaluate GCSF ability to improve pregnancy rate in women with history of implantation failure.

## Materials and methods

This randomized controlled trial (RCT) was performed at Yazd Research and Clinical Center for Infertility, Shahid Sadoughi University of Medical Sciences between October 2014 and February 2015. The study was approved by the Ethics Committee of the university. Informed written consent was obtained from all couples. 

Women between 20-40 yrs old with history of at least two implantation failures were included. Participants with sickle cell disease, chronic neutropenia, malignancy history, renal failure, congenital fructose intolerance, respiratory infection, endometriosis and sever male factor were excluded. A total of 90 eligible women were randomly allocated in two equal groups; intervention and control. The patient randomized by random allocation software. 

**Treatment protocol**

The patients received 150 IU/day Gonal f (Serono Labratories, Aubonne, Switzerland) starting on day 2 and 0.25 mg cetrorelix (Cetrotide; Asta Medica, Frankfurt, Germany) administered daily when one or more follicles reached 13-14 mm in diameter. The doses of recombinant FSH have been adjusted according to the ovarian response for both groups. Urinary hCG 10000 IU (Pregnyle, NV Organon, Oss, Netherlands) was administered when at least two leading follicles reached a mean diameter of 17 mm and the serum E_2_ concentration was >500 pg/mL. 

Transvaginal oocyte retrieval was scheduled 36 hr after hCG injection. 45 patients in interventional group received uterine infusion of 300 µg (0.5ml) recombinant human GCSF (300 µg, Zahravi Co., Tehran, Iran) by the use of IUI catheter after ovarian puncture under general anesthesia, while the standard treatment was continued in the 45 patients (control group) and did not receive GCSF treatment. Following oocyte retrieval, metaphase II oocytes were reviewed and good-quality embryos (Grade A: uniform or slightly uneven with fragmentation of <10%; Grade B: the blastomeres size uniform or non-uniform, fragmentation amount of 10-20%) were transferred on day 3 for all patients. 

Following embryo transfer (ET), all patients received Cyclogest® vaginal pessaries (Cox Pharmaceuticals, Barnstaple, UK) 400 mg twice daily until menstruation or for 8 wks after ET procedure in case of clinical pregnancy. Clinical pregnancy was defined as the presence of gestational sac with fetal heartbeat by ultrasound 4 wks following the ET. The implantation rate was defined as the ratio of gestational sac determination on transvaginal ultrasonography to the number of transferred embryos. 

**Statistical analysis**

All of statistical analysis was done by SPSS 20 (SPSS, Chicago, IL). With 95% confidence level, power of 80%, pR1R=20%, pR2R=45% and the sample size=45 in each group was considered. The normal distribution of data was checked. The normal distribution of data was checked. Mean±SD were calculated for descriptive analysis. Independent t-test and ^2^ were used. The statistical significances considered as p˂0.05. All of analyses were based on intention to treat analysis.

## Results

**Figure 1:**
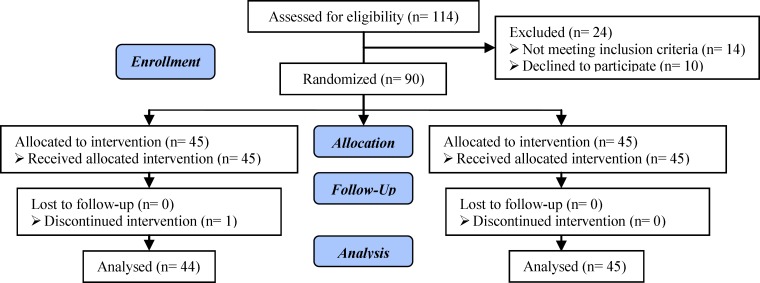
Consort flow chart

Totally 90 patients were included in this study (Figure 1). The mean age of participants was 31.95±4.71 years old (32.55±4.61 in GCSF and 31.75±5.16 in control group). The mean of FSH level in third day was 7.14±4.16 in GCSF group and 5.17±2.31 in controls. Demographic characteristics of participants and ART characteristics are shown in tables I and II respectively. There were no significant differences in female age, infertility duration, third day FSH, number of ART cycles, number of embryos and endometrial thickness between groups (p>0.05). The pregnancy rate in GCSF group was improved significantly. Thirteen women (28.88%) in GCSF group and 6 (13.3%) in control group were clinical pregnant (p=0.043).

**Table I:** demographic characteristics of participants

**Variables**	**GCSF group**	**Control group**	**The significance**
Age (year)	32.55 ± 4.61	31.75 ± 5.16	0.44
Infertility duration (year)	9.2 ± 4.31	8.6 ± 7.22	0.63
Num of pervious failed ART	2.57 ± 1.69	3.41 ± 1.54	0.055
Third day FSH (m IU/ml)	7.14 ± 4.16	5.17 ± 2.31	0.059

*Data are presented as mean±SD.

# Student *t*-test

*#Basal FSH level (day 3 FSH) (IU/L)

**Table II:** Cycle characteristics of participants

**Variables**		**GCSF group**	**Control group**	**p-value**
Gonal-F (IU)^*^^#^		1791 ± 546.75	1647 ± 570.75	0.168
Cycle duration (day) ^*^^#^		11.59 ± 2.46	10.75 ± 1.46	0.056
Num of retrieved oocyte ^*^^#^		11.36 ± 6.03	14.53 ± 7.34	0.029
Num of oocyte (M II) ^*^^#^		9.6 ± 6.6	12.46 ± 6.28	0.033
Num of embryos ^*^^#^		7.04 ± 5.71	7.02 ± 4.29	0.983
Num of transferred embryos ^*^^#^		2.11 ± 0.77	2.35 ± 0.71	0.123
Embryo quality^*^^$^				
	A	27 (61.4%)	26 (57.8%)	0.830
	B	17 (38.6%)	19 (42.2%)	
Endometrial thickness in in hCG triggering day (mm) ^*^^#^		9.13 ± 1.45	8.79 ± 1.65	0.306
Implementation rate^**^^#^		16.67%	5.04%	0.0151
Clinical pregnancy^**^^$^		13 (28.88%)	6 (13.3%)	0.043

Data presented as mean±SD. (n=45)

$ Data presented as n (%)

# Data presented as mean±SD.

* Comparison was done by Independent t-test.

** The comparison was done by ².

## Discussion

The endometrial receptivity and thickness play essential role in implantation phase (18, 20, 21). Based on our study, clinical pregnancy rate was improved by GCSF intrauterine infusion on ovarian puncture day. similar to previous studies that indicated G-CSF administration may have beneficial effect on clinical pregnancy outcome after ART our findings showed that intrauterine infusion significantly improves the clinical pregnancy rate (22). One of the first studies of GCSF was a prospective cohort study on 4 patients to evaluate the effect of GCSF on thin endometrium. These cohort findings represented GCSF is a new treatment of thin endometrium (18). 

After the pilot study Barad *et al* studied the GCSF effect on endometrium and clinical pregnancy rate in a parallel double blinded placebo-controlled clinical trial with 141 consecutive, unselected, consenting women with no history of renal disease, sickle cell disease, or malignancy who were undergoing IVF, in this study GCSF does not improve endometrial thickness or pregnancy rate (23). The other study on 37 patients with thin endometrium demonstrated that infusion of GCSF improves the endometrial thickness (24). Eftekhar *et al* also in an interventional study on 67 infertile women showed although GCSF did not improve endometrial thickness but significantly increased the pregnancy rate in women with thin endometrium (20). But most of the previous studies reported the GCSF role on endometrial thickness improvement (24, 25). The useful role of GCSF on implantation was overlooked. 

In spite of lesser follicles and metaphase II oocytes in GCSF group (statistics significance), implantation and pregnancy rate were higher than control. Our study findings showed that clinical and chemical pregnancy rate were improved by GCSF intrauterine infusion on the day of ovarian puncture. The point of GCSF effect may be endometrial-embryo interaction which should be added to many positive influences of GCSF such as effect on ovarian function, granulose cell function, human decidual macrophages, ovulation and reduction of unexplained repeated abortion (14, 16). The most important limitation of the rare studies about the role of GCSF on implantation was limited sample size but in our study this limitation was satisfied. Although the mechanism of intrauterine infusion of GCSF is not found by details but since intrauterine GCSF decreases CD16 and CD56 and also increases LIF significantly, so the chances of getting pregnant will be improved (23). As noted in some studies exogenous GCSF intrauterine infusion as chemical and mechanical stimuli can improve the implantation and pregnancy outcomes. The considered mechanism is that GCSF may induce secretion of endogenous cytokines activating the endocrine pathway (14, 20, 23-25). Although our sample size was adequate but there were no blinding in our study. 

## Conclusion

As a conclusion intrauterine infusion of GCSF in infertile women with history of implantation failure is an effective treatment and can improve the pregnancy outcome.

## Note

***Registration ID in IRCT: IRCT201510086420N15***
